# Previous Influenza Infection Exacerbates Allergen Specific Response and Impairs Airway Barrier Integrity in Pre-Sensitized Mice

**DOI:** 10.3390/ijms22168790

**Published:** 2021-08-16

**Authors:** Kevin Looi, Alexander N. Larcombe, Kara L. Perks, Luke J. Berry, Graeme R. Zosky, Paul Rigby, Darryl A. Knight, Anthony Kicic, Stephen M. Stick

**Affiliations:** 1 Wal-yan Respiratory Research Centre, Telethon Kids Institute, Nedlands 6009, Australia; Kevin.Looi@telethonkids.org.au (K.L.); kara.perks@telethonkids.org.au (K.L.P.); luke.berry@telethonkids.org.au (L.J.B.); anthony.kicic@telethonkids.org.au (A.K.); stephen.stick@health.wa.gov.au (S.M.S.); 2 Occupation, Environment and Safety, School of Population Health, Curtin University, Perth 6845, Australia; 3 Menzies Institute for Medical Research, College of Health and Medicine, University of Tasmania, Hobart 7001, Australia; graeme.zosky@utas.edu.au; 4 Tasmanian School of Medicine, College of Health and Medicine, University of Tasmania, Hobart 7001, Australia; 5 Centre for Microscopy, Characterisation and Analysis (CMCA), University of Western Australia, Nedlands 6009, Australia; paul.rigby@uwa.edu.au; 6 School of Biomedical Sciences and Pharmacy, University of Newcastle, Callaghan 2308, Australia; dknight2@providencehealth.bc.ca; 7 Priority Research Centre for Asthma and Respiratory Disease, Hunter Medical Research Institute, Newcastle 2305, Australia; 8 Department of Anaesthesiology, Pharmacology and Therapeutics, University of British Columbia, Vancouver, BC V6T 1Z4, Canada; 9 Centre for Cell Therapy and Regenerative Medicine, University of Western Australia, Nedlands 6009, Australia; 10 Department of Respiratory and Sleep Medicine, Perth Children’s Hospital, Perth 6009, Australia

**Keywords:** house dust mite, lung function, BALB/c mice, influenza, tight junctions, epithelial barrier integrity

## Abstract

In this study we assessed the effects of antigen exposure in mice pre-sensitized with allergen following viral infection on changes in lung function, cellular responses and tight junction expression. Female BALB/c mice were sensitized to ovalbumin and infected with influenza A before receiving a second ovalbumin sensitization and challenge with saline, ovalbumin (OVA) or house dust mite (HDM). Fifteen days post-infection, bronchoalveolar inflammation, serum antibodies, responsiveness to methacholine and barrier integrity were assessed. There was no effect of infection alone on bronchoalveolar lavage cellular inflammation 15 days post-infection; however, OVA or HDM challenge resulted in increased bronchoalveolar inflammation dominated by eosinophils/neutrophils or neutrophils, respectively. Previously infected mice had higher serum OVA-specific IgE compared with uninfected mice. Mice previously infected, sensitized and challenged with OVA were most responsive to methacholine with respect to airway resistance, while HDM challenge caused significant increases in both tissue damping and tissue elastance regardless of previous infection status. Previous influenza infection was associated with decreased claudin-1 expression in all groups and decreased occludin expression in OVA or HDM-challenged mice. This study demonstrates the importance of the respiratory epithelium in pre-sensitized individuals, where influenza-infection-induced barrier disruption resulted in increased systemic OVA sensitization and downstream effects on lung function.

## 1. Introduction

Asthma is a multifactorial, complex disease of the airways affecting both children and adults. Although the specific etiology is not clear, genetic susceptibility and exposure to various environmental triggers, including respiratory viruses and airborne allergens, are known to lead to its characteristic features of airway inflammation and excessive bronchoconstriction [1]. In recent years, the role of the airway epithelium in the pathogenesis of asthma has been extensively studied, with factors such as its immune functions [2] and its inherent abnormalities in asthmatic patients [3] being key foci. This is understandable, as the airway epithelial layer is the frontline of defense against aeropathogens through the establishment and maintenance of a physical barrier [4,5,6]. Due to this role, an altered epithelial layer is thought to be a potent driver of allergic asthma development and severity [7]. The integrity of this layer is dependent upon junctional complexes between adjacent epithelial cells which act synergistically and dynamically to provide the initial protective interface between the internal environment of the lungs and the external milieu of pathogens, aeroallergens and other toxicants. The junctional complexes include tight junctions, adherens junctions, gap junctions, desmosomes and connexins. Of importance are the tight junctions (TJ), which are typically located at the apical borders of adjacent epithelial cells where they serve to regulate the movement of ions and solutes as well as to prevent unwanted migration of pathogens and their products to the subepithelial space [8]. In addition, they play a vital role in the maintenance of a healthy and intact airway epithelium [9]. Damage to the epithelial barrier, including disruption in the TJ proteins via environmental pollutants, aeroallergens or respiratory pathogens, has been shown to increase the paracellular traffic of pathogenic molecules into the lung interstitium [5].

**Figure 1:**
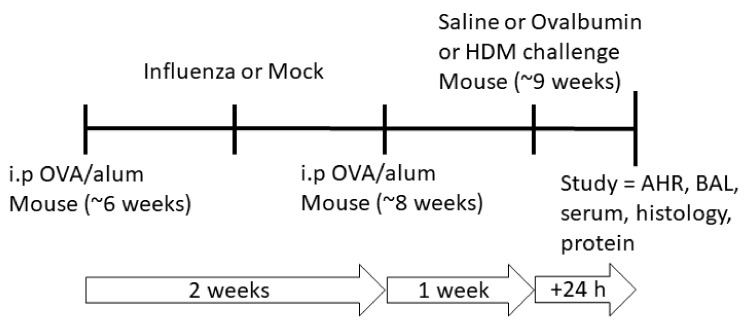
Adult mice were initially sensitized to chicken egg OVA in aluminum hydroxide (alum) and randomly assigned to a treatment group. One week later, mice were either mock-infected or infected with influenza/Mem71. Seven days post infection, mice received a second OVA/alum sensitization and 1 week later were challenged with either saline, OVA or house dust mite (HDM). All in vivo studies and sample collections were performed 24 h following saline, OVA or HDM challenge.

Although it has been recognized for many years that respiratory pathogens such as viruses can trigger asthma exacerbations, a growing body of evidence has shown that most acute episodes of asthma are associated with human rhinoviruses (HRV), respiratory syncytial viruses (RSV), metapneumovirus and influenza A virus (IAV) [10,11,12,13]. Infection with these respiratory viruses is associated with 80–85% of acute asthma exacerbations in children and 45% in adults [14]. In addition, these respiratory viruses are also known to disrupt the epithelial barrier and alter innate immune defenses [4,5,6]. However, there is a paucity of data on whether respiratory viral infection and the associated epithelial barrier disruption in susceptible (pre-sensitized to allergen) individuals further facilitates allergic sensitization and subsequently the development of an allergic airways disease phenotype. Additionally, it is unknown whether responses are antigen specific. To address this gap in knowledge, we used a combination of murine models to test the hypothesis that respiratory viral infection in individuals previously sensitized to allergen results in epithelial barrier dysfunction, augmenting antigen-specific allergic sensitization and subsequently resulting in an allergic airways disease phenotype. Importantly, our study differs from others in that we explored outcomes in mice pre-sensitized with allergen, in order to explore the impacts of respiratory viral infection on this subset of susceptible individuals. We also tested whether responses were antigen specific by systemically sensitizing individual mice with ovalbumin, and then challenging them with either the same allergen, or a different allergen (house dust mite) after the respiratory viral infection had cleared (Figure 1). Therefore, this study aimed to: (i) assess cellular and physiological responses following influenza infection and the development of allergic airway disease in pre-sensitized mice; and (ii) determine the relationship between viral infection, epithelial barrier impairment and development of an allergic airways disease phenotype.

## 2. Results

The main goal of this study was to assess whether a respiratory viral infection in a pre-sensitized individual would impair epithelial barrier function, thereby facilitating subsequent allergic sensitization and the effects of antigen-specific challenge. Our results show that influenza infection in an individual previously sensitized to OVA was associated with impaired epithelial barrier integrity and higher OVA-specific serum IgE. Impaired barrier integrity may potentially provide easier access to the airway smooth muscle for both inhaled allergens (OVA and HDM) and bronchoconstricting agents such as methacholine. Previous influenza infection did not lead to significant changes in pulmonary cellular inflammation, which was more significantly impacted by challenge allergen. Finally, mice pre-sensitized with OVA, infected with influenza and then challenged with OVA displayed the highest airway hyperresponsiveness, indicating that functional impairment can be a downstream consequence in this model.

### 2.1. Mass

There were significant independent effects of both infection (*p* = 0.009) and challenge (*p* = 0.043) on the weights of mice on the day of study; however, there were no interactions between these factors (*p* = 0.098). Mice previously infected with influenza (17.47 + 1.04 g) weighed significantly less than uninfected mice (18.32 ± 1.46 g). Mice challenged with OVA (17.33 ± 1.20 g) weighed significantly less than mice challenged with saline (18.18 ± 1.57 g), or HDM (18.18 ± 1.02 g).

### 2.2. Cellular Inflammation

**Figure 2:**
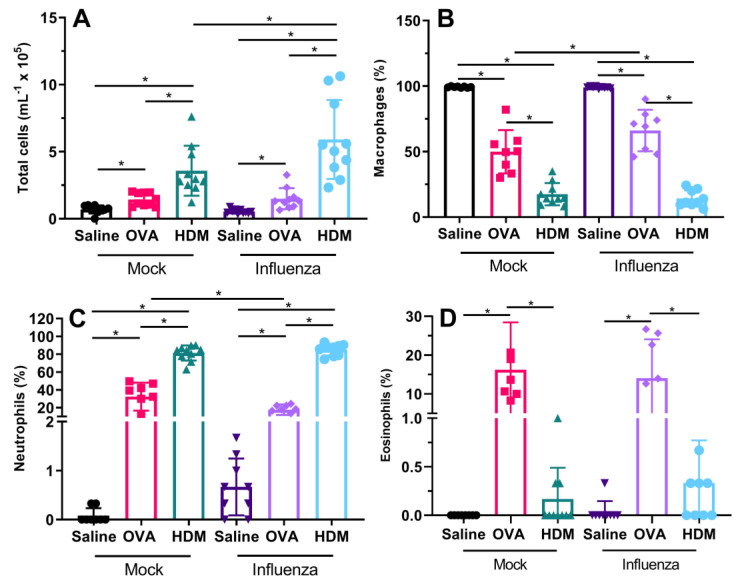
Mice which were challenged with either saline, ovalbumin or HDM showed similar response patterns in total cell counts between mock- or influenza-infected groups (**A**). Cellular inflammation was almost entirely composed of macrophages in saline-challenged mice in both groups. In OVA-challenged mice, the composition of inflammatory cells was mainly macrophages, followed by neutrophils and eosinophils. In contrast, cellular inflammation in HDM-challenged mice was predominantly neutrophils (**C**), followed by macrophages (**B**) and trace levels of eosinophils (**D**). Data are mean ± SD, * indicates significant difference between groups; *p* < 0.05.

There was no effect of previous influenza infection on total numbers of cells in BAL for saline-challenged (*p* = 0.056) or OVA-challenged (*p* = 0.996) mice; however, influenza-infected and HDM-challenged (IH) mice had significantly more total cells in their BAL than mock-infected and HDM-challenged (MH) mice (*p* = 0.009) (Figure 2A). With respect to the percentage of cells that were macrophages, there was no effect of influenza infection for saline-challenged (*p* = 0.953) or HDM-challenged (*p* = 0.481) mice; however, influenza-infected and OVA-challenged (IO) mice had a significantly greater percentage of macrophages in their BAL compared with mock-infected and OVA-challenged (MO) mice (*p* = 0.002; Figure 2B). There was also a significant interaction between challenge allergen and infection for the percentage of inflammatory cells that were neutrophils (*p* < 0.001; Figure 2C). IO mice had a significantly lower percentage of BAL neutrophils compared with MO mice (*p* < 0.001); however, there was no significant difference between IH and MH (*p* = 0.330) or influenza-infected and saline-challenged (IS) and mock-infected and saline-challenged (MS) mice (*p* = 0.882). There was also a challenge specific response with respect to eosinophils (Figure 2D); OVA-challenged mice had significantly higher percentage of eosinophils in their BAL than saline- or HDM-challenged mice (*p* < 0.001 in both cases), regardless of whether they were previously infected with influenza or not. Previous influenza infection did not impact the percentage of BAL eosinophils (*p* = 0.705).

### 2.3. Serum IgE

**Figure 3:**
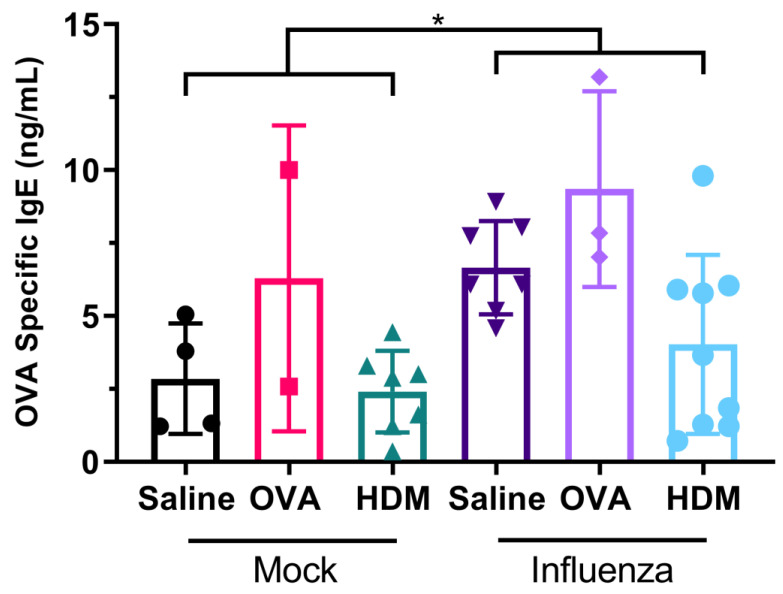
Mice pre-sensitized with OVA and previously infected with influenza had significantly higher levels of OVA-specific IgE in their serum than mice not previously infected with influenza. There was no significant effect of challenge antigen on OVA-specific IgE. Data are mean ± SD, * indicates significant difference between groups; *p* < 0.05.

Influenza infection resulted in an increase in OVA-specific IgE regardless of challenge (*p* = 0.020; Figure 3). Overall, mice previously infected with influenza had almost double the OVA-specific IgE in their serum compared with uninfected mice (5.84 ± 3.20 ng/mL compared with 3.14 ± 2.48 ng/mL). There was no significant effect of challenge allergen type on OVA-specific IgE for uninfected (*p* > 0.593 in all cases) or previously infected mice (*p* > 0.176 in all cases).

### 2.4. Lung Mechanics and Responsiveness to Methacholine

There was no effect of previous influenza infection on any parameter of lung function at functional residual capacity (FRC; airway resistance *p* = 0.613, tissue damping *p* = 0.739, tissue elastance *p* = 0.608; data not shown). There was also no effect of challenge with saline, OVA or HDM on any parameter of lung function at FRC (*p* > 0.127 in all cases). There was no interaction between previous infection and challenge allergen for any parameter of lung function at FRC (*p* > 0.092 in all cases).

**Figure 4:**
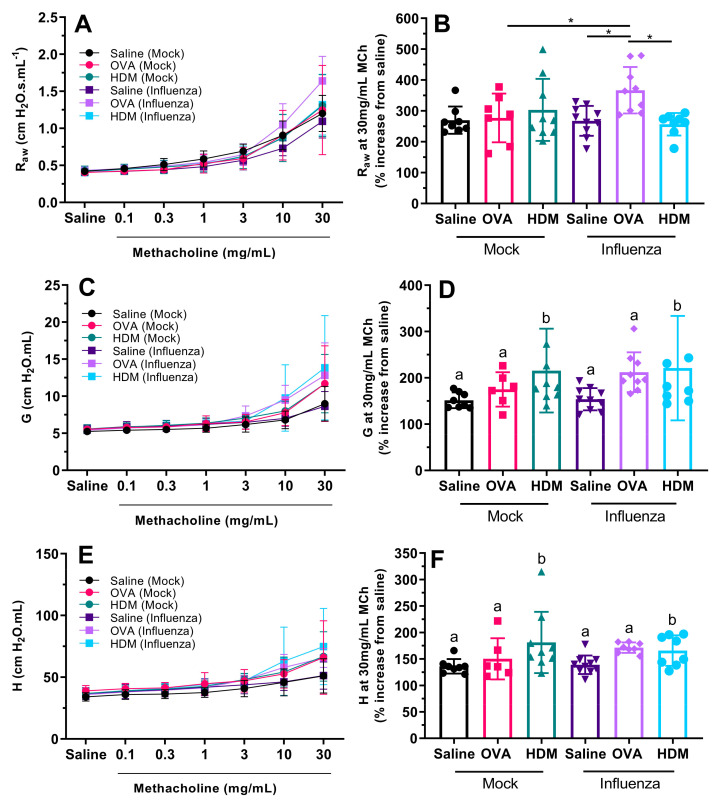
Mice which were sensitized, infected with influenza and subsequently challenged with OVA were the most responsive to methacholine in terms of airway resistance (**A**,**B**). Airway resistance at the maximum dose of MCh in mice which were challenged with saline or HDM was not significantly different between mock- and influenza-infected groups (**B**). Previous influenza infection had no significant effect on either tissue damping (**C**,**D**) or tissue elastance (**E**,**F**); however, mice challenged with HDM were more responsive in these parameters compared with mice challenged with either OVA or saline. Data are mean ± SD, * indicates significant difference between groups and different letters indicate significant differences between HDM and other treatments; *p* < 0.05.

There was a significant interaction between previous influenza infection and challenge type with respect to airway resistance at the maximum dose of MCh (Figure 4A,B; *p* = 0.026). For mice previously infected with influenza, those subsequently challenged with OVA (IO) were significantly more responsive than those challenged with saline or HDM (*p* = 0.009 in both cases). There was no effect of challenge type on airway hyperresponsiveness for mice not previously infected with influenza (*p* > 0.703 in all cases). Importantly, there was a significant effect of previous influenza infection with respect to airway resistance at the maximum dose of MCh for mice challenged with OVA (*p* = 0.011), but not for mice challenged with saline (*p* = 0.902) or HDM (0.235); IO mice showed a significantly greater degree of airway hyperresponsiveness than MO mice (*p* = 0.011).

For tissue damping (G; Figure 4C, D) there was a significant effect of challenge type (*p* = 0.016) but not previous influenza infection (*p* = 0.423) with respect to responsiveness to MCh. HDM-challenged mice were more responsive than saline- or OVA-challenged mice, regardless of previous infection status (*p* = 0.014). The same was true for tissue elastance (H; Figure 4E, F), whereby there was a significant effect of challenge type (*p* = 0.007) but not previous influenza infection (*p* = 0.313), with HDM-challenged mice being more responsive than either saline- or OVA-challenged mice, regardless of previous infection (*p* = 0.006).

### 2.5. Barrier Integrity

**Figure 5:**
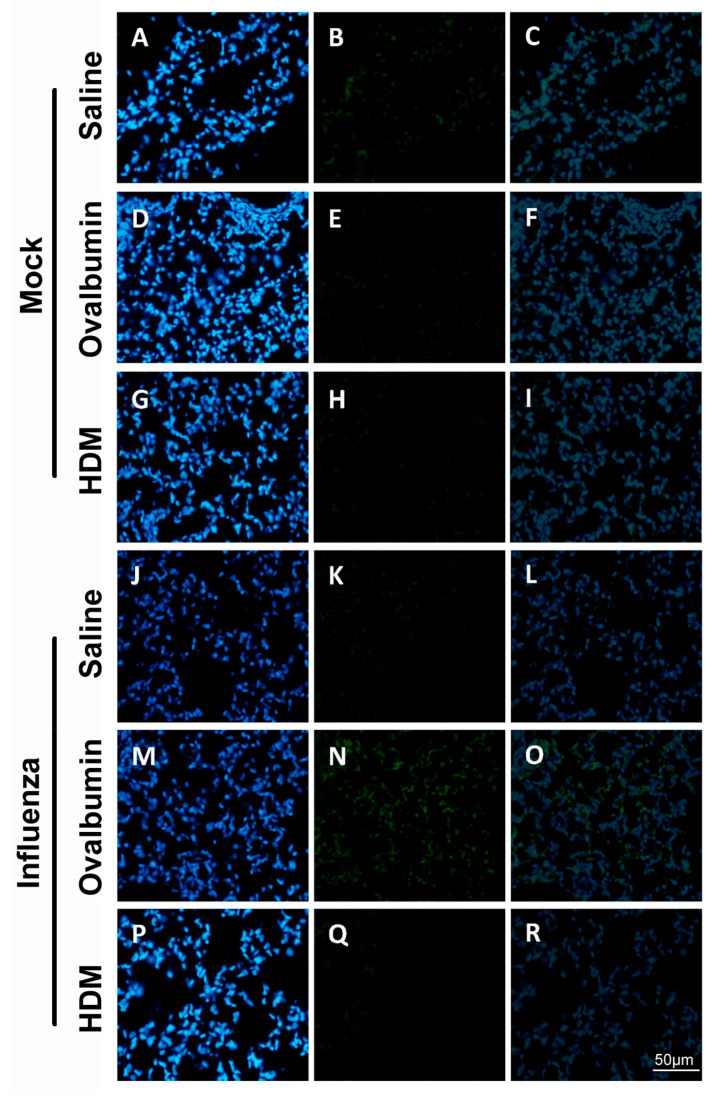
Nuclei (Blue) and claudin-1 (Green) staining in lungs of mock-infected treatment challenged with saline (**A**–**C**); ovalbumin (**D**–**F**); and HDM (**G**–**I**). Decrease in staining intensity was observed in influenza-infected treatments challenged with saline (**J**–**L**) and HDM (**P**–**R**). However, a marked increase in staining intensity was noted in the influenza-infected treatment challenged with ovalbumin (**M**–**O**). All images were acquired at × 400 total magnification. Scale bar = 50 µm.

**Figure 6:**
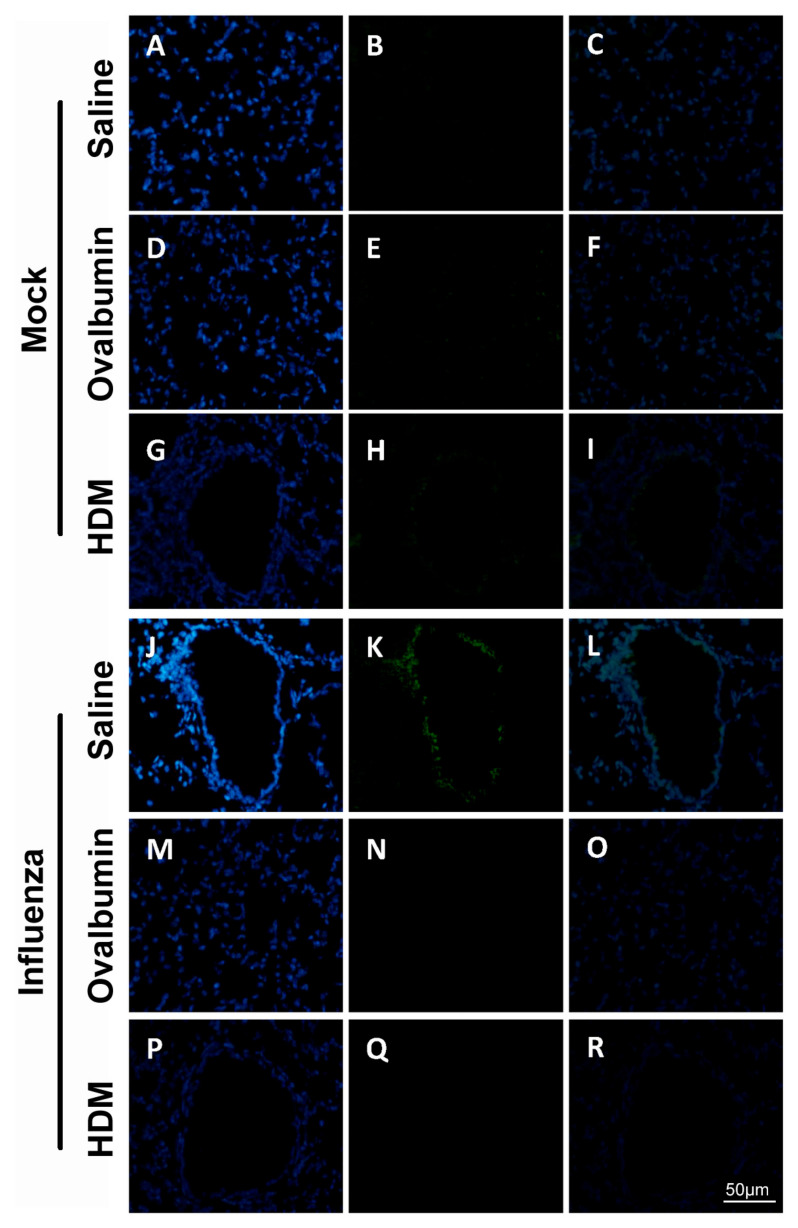
Nuclei (Blue) and occludin (Green) staining in lungs of mock-infected treatment challenged with saline (**A**–**C**); ovalbumin (**D**–**F**); HDM (**G**–**I**). A marked increase in staining intensity was noted in the influenza-infected treatment challenged with saline (**J**–**L**). Decrease in staining intensity was observed in the influenza-infected treatment challenged with ovalbumin (**M**–**O**) and HDM (**P**–**R**). All images were acquired at × 400 total magnification. Scale bar = 50 µm.

To investigate the direct in vivo effects of influenza infection on barrier integrity, we examined the TJs claudin-1 and occludin, key junctional proteins for the maintenance of epithelial integrity via immunohistochemical staining (Figure 5). We observed that basal claudin-1 staining within the mock-infected treatment challenged with saline, OVA or HDM (Figure 5A–I) was low, with the greatest intensity observed in mock-infected mice challenged with saline. However, a marked increase was noted in the influenza-infected mice, with the greatest increase in staining observed in mice previously infected with influenza and challenged with OVA (Figure 5N). Minimal staining was observed for occludin (Figure 6) within the similarly challenged mock-infected treatments (Figure 6A–I). Conversely, a marked increase was observed in mice previously infected with influenza and challenged with saline (Figure 6K), while minimal change in staining was observed for mice previously infected with influenza and challenged with OVA or HDM (Figure 6N,Q).

**Figure 7:**
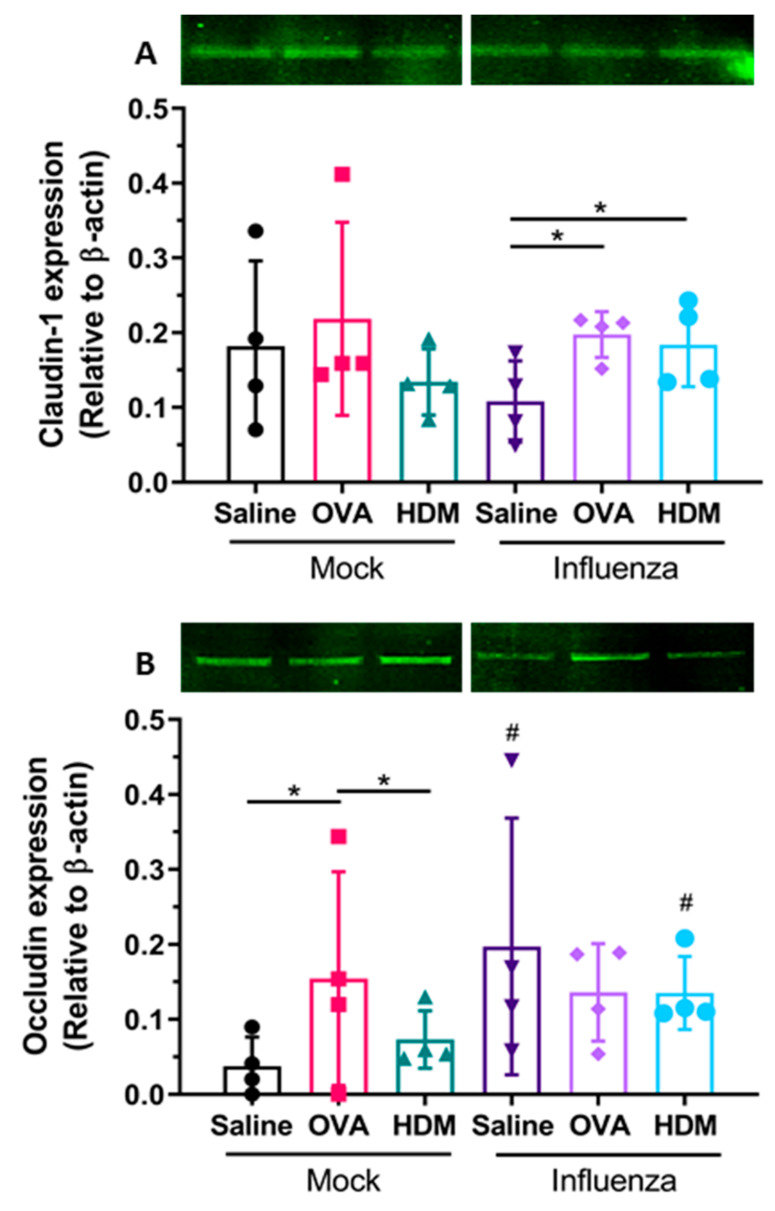
TJ protein expression in lungs of OVA/alum pre-sensitized mice after mock or influenza infection and following saline, ovalbumin or HDM challenge. Western blot and band densitometry analysis showed (**A**) claudin-1 expression was significantly higher in influenza-infected and ovalbumin- or HDM-challenged mice compared to saline; (**B**) occludin expression was significantly higher in mock-infected, ovalbumin-challenged mice compared to either saline- or HDM-challenged mice. Occludin expression was significantly higher in influenza-infected, saline- or HDM-challenged mice compared to their mock-infected counterparts. Data are mean ± SD, * statistical significance within either mock- or influenza-infected treatment (*p* < 0.05); ^#^ statistical significance compared to mock infected (*p* < 0.05).

We next investigated TJ protein expression of claudin-1 and occludin. Our data demonstrate no significant difference in claudin-1 protein expression levels in challenged mock-infected mice (Figure 7A—Mock). Conversely, a significant increase in claudin-1 expression was observed in mice previously infected with influenza and then challenged with OVA or HDM compared with mice challenged with saline (Figure 7A—Influenza; *p* < 0.05). When occludin expression was assessed, significantly increased expression was observed in mock-infected OVA-challenged mice compared with saline- or HDM-challenged mice (Figure 7B—Mock; *p* < 0.05). This was not observed in mice previously infected with influenza. However, when comparing occludin expression, a significant increase in protein expression was observed in previously infected saline- or HDM-challenged mice compared with mock-infected mice identically challenged (Figure 7B; *p* < 0.05).

## 3. Discussion

We investigated the effects of influenza infection on mice, pre-sensitized with OVA and subsequently challenged with saline, OVA or HDM in terms of epithelial barrier function and features of allergic airways disease (pulmonary inflammation, responsiveness to methacholine, etc.). The main novel outcome of this study was that in previously sensitized mice, influenza infection resulted in a disrupted epithelial barrier fifteen days post infection (after the acute virus-induced inflammation has resolved), and this was associated with the greatest degree of airway hyperresponsiveness (AHR; in terms of airway resistance). We suggest that influenza infection disrupts the epithelial barrier in pre-sensitized individuals, thus facilitating allergic sensitization, leading to exacerbation of an allergic airways disease phenotype. Importantly, the greatest functional deficits were seen in mice previously infected with influenza but who were also sensitized and challenged with the same allergen.

As mentioned, the key novel finding of our study was that OVA-specific IgE levels were significantly higher in mice previously infected with influenza, compared with those mock infected. In conjunction with our finding that infection with influenza results in the damage of tight junctions in mice, this is evidence that prior viral infection facilitates allergic sensitization. Some of these observations have been observed previously [15], whereby BALB/c mice infected with influenza A (H2N2/Kumamoto) had increased OVA-specific IgE in their serum, provided they were challenged with OVA during acute infection. However, and importantly in our study, mice were not challenged with allergen during acute infection (which is typically resolved in less than ten days [16,17]), but did receive their second systemic sensitization with OVA during this period. Conversely, RSV infection prior to OVA sensitization did not enhance production of OVA-specific IgE in BALB/c mice [18], suggesting responses are virus specific. In our model, this was followed by a significant increase in airway resistance in influenza-infected mice challenged with OVA when compared with influenza-infected mice challenged with saline.

We also found that previous influenza infection had limited effects on inflammatory response, with only HDM-challenged mice showing an increase in total cells in previously infected mice when compared with uninfected mice. Previously infected and OVA-challenged mice had a reduced proportion of neutrophils, balanced by a greater proportion of macrophages compared with non-infected OVA-challenged mice. With respect to our original hypothesis, we expected that influenza infection would facilitate allergic sensitization via epithelial barrier disruption; however, in terms of total pulmonary inflammation, this was only true for HDM-challenged mice. The observed inflammatory profile following influenza infection and allergen sensitization/challenge was expected, as we have previously shown that Mem71 infection induces a macrophage/neutrophil-dominated response [19], although in the current study, the increased inflammation has persisted for a longer period of time. Eosinophilic inflammation was anticipated following OVA challenge, it is a key feature of the OVA sensitization/challenge model in BALB/c mice [20]. In HDM-challenged mice following influenza infection, neutrophil levels were significantly higher followed by macrophages with minimal or no eosinophils detected. Again, this was expected, as we and others have previously shown that sensitization and challenge with HDM extracts high in proteases results in a neutrophil-dominated inflammatory response [21,22].

Even though there was a lack of OVA-specific inflammatory increases in previously infected mice, the greatest airway hyperresponsiveness that we measured was in previously infected mice challenged with OVA. We expected OVA sensitization and challenge to induce AHR; however, this was not the case in mock-treated mice (Figure 4A–B). Only OVA-challenged mice that had the additional insult of influenza infection displayed AHR (relative to those challenged with saline or HDM, or mock-infected mice). This was unexpected for mock-infected mice as we employed a well-established allergic airways disease model [20,23]. It is possible that subtle differences in the age of the mice may be responsible, since we commenced the OVA sensitization protocol when mice were 6 weeks of age, 2 weeks younger than in Zosky et al. (2008). Eight-week-old mice are generally considered adult, and have a fully developed immune system, while at six weeks of age, immune maturation (in terms of T cell responses and T and B lymphocyte production) is still occurring [24,25].

Next, we observed that infection with influenza resulted in detectable damage to tight junctions in mice, fifteen days post infection. Specifically, staining intensity of claudin-1 of the respiratory epithelial cells was greater in previously infected groups, compared with mock-infected mice, whereas changes in occludin expression were only observed in OVA- or HDM-challenged mice. Of all the TJ proteins, claudin-1 appears the most strongly expressed by large and small airway epithelial cells [26]. Increased claudin-1 expression following influenza infection and OVA or HDM challenge in comparison to saline was observed within our study despite intrinsic expression of claudin-1 within the airway epithelium being very low [27]. In contrast, strong occludin expression within the airway epithelium has been previously reported [28]; however, the present data demonstrate a loss in protein expression following influenza infection and OVA or HDM challenge. This suggests a possible association with influenza infection and sensitization where a compensatory effect of claudin-1 is seen as occludin expression is lost. Furthermore, when interpreted collectively, our data indicate that damage to the epithelial TJs is strongly associated with increased sensitization as serum OVA-specific IgE was similarly detected in all previously infected treatment groups at greater levels than in their mock-treated counterparts. However, there was no significant association between the damage to epithelial TJ and cellular inflammation in our present study.

Several possible mechanisms have been suggested which could result in the impairment of TJ in epithelial cells. However, although contrasting, most evidence at present has demonstrated that epithelial TJ disruption can be directly attributed to the virus infection itself [29,30,31]. More specifically, it has been shown by Golebiewski and colleagues that some highly pathogenic strains of influenza, such as H5N1, can bind to proteins essential to TJ formation and actively disrupt the formation of the TJ complex [31]. Thus, it is highly likely that components of the influenza virus are able to disrupt claudin-1 expression indirectly through binding and the subsequent sequestration of the binding pairs.

Previous work has also demonstrated changes in the epithelial TJ complexes [29] following influenza infection in vitro. Thus, it is possible that infection with influenza results in the dysregulation of barrier integrity more severely or for a longer duration, which could potentially facilitate allergic sensitization leading to an increased susceptibility and eventual development of an allergic asthma phenotype. We have also previously demonstrated that female BALB/c mice infected with influenza displayed persistent lung function deficits through to adulthood while male mice similarly infected recovered completely [19]. However, in the current study, we only assessed female BALB/c mice and thus, our current data are insufficient to address the potential impact of sex-based differences in epithelial permeability. Furthermore, previous in vitro work has also demonstrated the potential for a reduction in other TJ proteins following influenza infection in addition to claudin-1 and occludin assessed in the present study; nevertheless, our data have demonstrated the potential for influenza infection to cause damage in TJ complexes of mouse pulmonary airway epithelial cells.

In conclusion, the present study explores a model that encompasses the use of relevant aeroallergens capable of inducing an allergic airways response, with a ubiquitous respiratory pathogen in the form of influenza, to assess the impact of infection on barrier impairment in pre-sensitized mice. Data presented demonstrate alterations in epithelial integrity following influenza infection in pre-sensitized mice, leading to an increase in systemic allergic sensitization, and subsequent alterations in lung function. More broadly, these findings provide additional evidence that viral infection in pre-sensitized mice can lead to exacerbation of an allergic airways disease phenotype, but more importantly, suggest that pre-sensitized mice, previously infected with influenza and subsequently sensitized and challenged with the same allergen, display the most severe changes in lung function.

## 4. Materials and Methods

### 4.1. Ethics Statement

This project was approved by the Telethon Kids Institute Animal Ethics Committee (AEC #250) and was carried out in strict accordance with the Australian code for the care and use of animals for scientific purposes 8th edition (2013), with all efforts made to minimize animal suffering.

### 4.2. Animals

Female BALB/c mice (Animal Resources Centre; Murdoch, WA, Australia) were housed within individually ventilated cages (Sealsafe, Techniplast, Buguggiate, Italy) on non-allergic, dust-free bedding (Shepards Specialty Papers, Chicago, IL, USA). The mice were maintained on a 12:12 h light:dark cycle and provided with an allergen-free diet (Specialty Feeds, Glen Forrest, WA, Australia) and water *ad libitum*.

### 4.3. Virus and Infection

In this study we used the “Mem71” strain of influenza A. This H3N1 strain is a reassortant of A/Memphis/1/71 (H3N2) and A/Bellamy/42 (H1N1) [32]. The virus was propagated, purified and virus concentration assessed as previously described [33]. This strain of influenza A is of intermediate virulence [16] in which viral replication peaks three days post infection [17]. By seven days post infection, viral titers are below the limit of detection of plaque assays [17], indicating that viral replication and acute infection have resolved.

### 4.4. OVA/Alum and House Dust Mite Sensitization, Virus Infection and Treatment Groups

Adult (6-week-old) female mice were systemically sensitized using a well-established model of allergic airways disease [23], during which mice were also infected with influenza. Mice were first sensitized by intraperitoneal (i.p) administration of 20 µg of chicken egg OVA (Sigma) in 200 µL of aluminum hydroxide (alum; Serva) on day 0 and randomly assigned to a treatment group. Seven days later, mice were intranasally (i.n) infected with influenza/Mem71 (~10^4.5^ plaque-forming units; pfu) or the supernatant of uninfected Madin-Darby canine kidney (MDCK) cells diluted in Virus Production Serum Free Medium (VP-SFM; Gibco, Mulgrave, VIC, Australia) (day 7) under light methoxyfluorane anesthesia. Seven days post infection (day 14), mice received a second sensitization i.p. injection of OVA/alum and 1 week after the second OVA/alum sensitization (day 21), mice were challenged with a 30 min 1% OVA or saline aerosol or 100 µg house dust mite (HDM) protein in 50 µL saline via intranasal inoculation (Figure 1) as per previously published methods [21,23]. Twenty-four hours after challenge (day 22), mice were anesthetized for lung function assessment prior to being sacrificed and samples were collected for various assays as indicated below. The data and sample collection timepoint is 15 days post infection, well after peak viral loads and viral-induced inflammation have resolved. Therefore, we had six treatment groups, each with n = 10: influenza + OVA challenge (IO), influenza + HDM challenge (IH), influenza + saline challenge (IS), mock infection + OVA challenge (MO), mock infection + HDM challenge (MH) and mock infection + saline challenge (MS).

### 4.5. Lung Function and Responsiveness to Methacholine

All in vivo studies were performed 24 h after challenge. This timing was based upon our previous work [21,34]. The mice were surgically prepared and lung function at functional residual capacity (FRC) and responsiveness to methacholine were assessed using the forced oscillation technique (FOT) as previously described [19].

### 4.6. Measurement of Cellular Inflammation and Cytokines

At the conclusion of lung function assessment, bronchoalveolar lavage (BAL) fluid was collected by washing 0.5 mL of saline in and out of the lungs three times via the tracheal cannula of each mouse. Lavage samples were processed for total and differential cell counts as previously described [35].

### 4.7. Serum Antibodies

Following in vivo measurements, whole blood was collected via cardiac puncture, centrifuged at 2000 rpm for 15 min and serum was obtained for analysis of OVA-specific IgE by ELISA. Levels of OVA-specific IgE were measured as per the manufacturer’s instructions (BioLegend, San Diego, CA, USA).

### 4.8. Immunohistochemistry of Tight Junctional Proteins

Paraffin-embedded, formalin-fixed sections were initially deparaffinized, rehydrated and subjected to either heat- or protease-induced epitope retrieval process. Slides were then cooled to room temperature (RT) for 10 min followed by 3 washes in 1× TBS containing 0.1% (*v*/*v*) saponin. Sections were then blocked in 5% (*w*/*v*) BSA, 10% FBS (*v*/*v*), 0.1% (*v*/*v*) TritonX-100 and 0.1% (*v*/*v*) saponin in 1× TBS for 1 h at RT. After a second series of washes, sections were incubated with the primary antibodies to claudin-1 (1:100) and occludin (1:100), diluted in the blocking buffer solution and added to the slides which were then incubated overnight at 4 °C. The following day, sections were washed in 1× TBS with 0.1% (*w*/*v*) saponin (3 × 15 min/wash) for the detection of cytoplasmic markers. Fluorescent secondary antibodies were prepared in blocking buffer in the necessary concentration and were added to the slides which were further incubated overnight at 4 °C. The following day, slides were washed in 1× TBS with saponin. Once all primary and secondary antibodies had been used to stain the cells, the nucleus of the cells was counterstained with 4′, 6-diamidino-2-phenylindole (DAPI). Slides were incubated with DAPI (1:50,000) in 1× PBS for 10 min and then washed in 1× TBS (3 × 15 min/wash). Fluorescent mounting media was used to minimize fading and slides were visualized using a fluorescence microscope.

### 4.9. Western Blot

Protein was collected from cells by cell extraction buffer (CEB), quantitated by the bicinchoninic acid (BCA) assay and stored at -80 °C. Prior to Western blot analysis, the protein samples were thawed on ice to prevent protein degradation. A 10 µg protein sample was mixed with NUPAGE^®^ Lithium Dodecyl Sulphate (LDS, ThermoFisher Scientific, Waltham, MA, USA) buffer, NUPAGE^®^ reducing agent (ThermoFisher Scientific, Waltham, MA, USA) and ddH_2_O to make up a final volume of 20 µL. The samples were then heated for 10 min at 70 °C on a heating block for optimal denaturation before being loaded into a pre-cast 4–12% 1.0 mm Bis-Tris Plus polyacrylamide gel (Novex BOLT^®^, ThermoFisher Scientific, Waltham, MA, USA). Samples were then electrophoresed using a Novex BOLT^®^ Western blot apparatus (Life Technologies, Waltham, MA, USA) in MES SDS running buffer (ThermoFisher Scientific, Waltham, MA, USA) at a constant 200 V for 35 min at RT. A pre-stained protein ladder was run on all gels in addition to samples for reference purposes. After separation, proteins were transferred onto a PVDF membrane using a semi-dry transfer method on iBlot (Invitrogen^®^, (ThermoFisher Scientific, Waltham, MA, USA) system at 200 V for 7 min or a wet transfer method at a constant 230 mA for 2 h at 4 °C. Upon completion of protein transfer, the PVDF membrane (ThermoFisher Scientific, Waltham, MA, USA) was blocked for non-specific staining by using the LI-COR Odyssey Blocking Buffer (LI-COR Biosciences, Lincoln, NE, USA) for 60 min at RT. Membranes were then incubated, with gentle rocking, overnight at 4 °C with the primary antibodies to claudin-1 and occludin made up in LI-COR Odyssey Blocking Buffer. The membranes were then washed 3 times (15 min per wash) in 0.2% (*v*/*v* final) Tween-20 in 1×TBS solution at RT. After washing, membranes were incubated in the dark with respective IRDye^®^ secondary antibodies made up in a solution of LI-COR Odyssey Blocking Buffer (LI-COR Biosciences, Lincoln, NE, USA) with 2% (*v*/*v* final) Tween-20 diluent for 2 h at RT with gentle rocking. The membranes were then washed 3 times (15 min per wash) in 0.2% (*v*/*v* final) Tween-20 in 1×TBS solution followed by 2 times (10 min per wash) in 1×TBS alone. The membranes were then scanned using the LI-COR Odyssey infrared scanner (LI-COR Biosciences, Lincoln, NE, USA) at 680 nm and 800 nm channels. Bands of protein expression were quantified using the LI-COR Odyssey v.3.0 software (LI-COR Biosciences, Lincoln, NE, USA). The integrated intensity (I.I) of each band was then normalized to the I.I of the housekeeping protein, β-actin.

### 4.10. Statistics

Before statistical evaluation, all results were tested for population normality and homogeneity of variance and corrected via appropriate transformation where necessary. Unless otherwise stated, analyses were performed via two-way ANOVA with infection (i.e., previous influenza infection, or mock infection) and challenge allergen (OVA, HDM or saline) as factors and Holm–Sidak post hoc tests. SigmaPlot 14 (Systat Software, San Jose, CA, USA) and GraphPad Prism 8 (GraphPad Software, San Diego, CA, USA) software were used for data analyses. Data are reported as mean ± SD. *p* < 0.05 was considered significant.

## Data Availability

Data supporting our results can be obtained from the corresponding author upon request.
